# Hema-Functionalized Graphene Oxide: a Versatile Nanofiller for Poly(Propylene Fumarate)-Based Hybrid Materials

**DOI:** 10.1038/s41598-019-55081-2

**Published:** 2019-12-10

**Authors:** Eugeniu Vasile, Andreea M. Pandele, Corina Andronescu, Aida Selaru, Sorina Dinescu, Marieta Costache, Anamaria Hanganu, Matei D. Raicopol, Mircea Teodorescu

**Affiliations:** 1 Department of Science and Engineering of Oxide Materials and Nanomaterials, University Politehnica of Bucharest, 1-7 Polizu St., 011061 Bucharest, Romania; 2 Advanced Polymer Materials Group, University Politehnica of Bucharest, 1-7 Polizu St., 011061 Bucharest, Romania; 3 Department of Analytical Chemistry and Environmental Engineering, University Politehnica of Bucharest, 1-7 Polizu St., 011061 Bucharest, Romania; 4 Chemical Technology III, University of Duisburg-Essen, Carl-Benz-Straße 199, D-47057 Duisburg, Germany; 5 CENIDE Center for Nanointegration, University of Duisburg-Essen, Carl-Benz-Straße 199, D-47057 Duisburg, Germany; 6 Department of Biochemistry and Molecular Biology, University of Bucharest, 91–95 Splaiul Independentei, 050095 Bucharest, Romania; 7 Department of Organic Chemistry, Biochemistry and Catalysis, University of Bucharest, 90-92 Şos. Panduri, 050657 Bucharest, Romania; 8 Costin Nenitzescu” Department of Organic Chemistry, University Politehnica of Bucharest, 1-7 Polizu St., 011061 Bucharest, Romania; 9 Department of Bioresources and Polymer Science, University Politehnica of Bucharest, 1-7 Polizu St., 011061 Bucharest, Romania

**Keywords:** Biomaterials, Graphene

## Abstract

Poly(propylene fumarate) (PPF) is a linear unsaturated polyester which has been widely investigated for tissue engineering due to its good biocompatibility and biodegradability. In order to extend the range of possible applications and enhance its mechanical properties, current approaches consist in the incorporation of various fillers or obtaining blends with other polymers. In the current study we designed a reinforcing agent based on carboxylated graphene oxide (GO-COOH) grafted with 2-hydroxyethyl methacrylate (GO@HEMA) for poly(propylene fumarate)/poly(ethylene glycol) dimethacrylate (PPF/PEGDMA), in order to enhance the nanofiller adhesion and compatibility with the polymer matrix, and in the same time to increase the crosslinking density. The covalent modification of GO-COOH was proved by Fourier-transform infrared spectroscopy (FT-IR), X-ray photoelectron spectroscopy (XPS), thermogravimetric analysis (TGA) and Raman spectroscopy. The mechanical properties, water uptake capacity, morphology, biodegradability, mineralization and *in vitro* cytotoxicity of PPF/PEGDMA hybrid materials containing GO@HEMA were investigated. A 14-fold increase of the compressive modulus and a 2-fold improvement in compressive strength were observed after introduction of the nanofiller. Moreover, the decrease in sol fraction and solvent swelling in case of the hybrid materials containing GO@HEMA suggests an increase of the crosslinking density. SEM images illustrate an exfoliated structure at lower nanofiller content and a tendency for agglomeration at higher concentrations. Finally, the synthesized hybrid materials proved non-cytotoxic to murine pre-osteoblast cells and induced the formation of hydroxyapatite crystals under mineralization conditions.

## Introduction

The development of new scaffold materials with improved mechanical properties and biodegradability is of great importance in the field of tissue engineering. Until now, both inorganic and organic materials were investigated, and biodegradable synthetic polymers emerged as the most promising candidates for orthopedic applications^1,2^. Poly(propylene fumarate) (PPF) is an unsaturated linear polyester that can be easily crosslinked either thermally or with photoinitiators^3–5^. Even though a study revealed that uncrosslinked poly(propylene fumarate) exhibits a cytotoxic effect towards fibroblasts^6^, fully crosslinked PPF networks as well as their non-enzymatic degradation products are generally considered biocompatible^7^. As drawbacks, PPF is difficult to handle since it is a highly viscous liquid at room temperature and has poor intrinsic mechanical properties, making it unsuitable for load-bearing orthopedic applications. For extending its range of applications, current approaches focus on mixing PPF with other polymers^8^ or various nanofillers^9^ which improve the mechanical properties. In addition, PPF is often mixed with inorganic materials including hydroxyapatite^10^, calcium carbonate or calcium sulfate^11^, β-tricalcium phosphate^12^ and titania^13^. Several other studies report the incorporation of carbonaceous nanostructures such as carbon nanotubes, functionalized graphene oxide, graphene oxide nanoribbons or nanoplatelets, and fullerenes in PPF-based nanocomposite materials^8,9,14–16^.

The properties of PPF-based composite materials can also be tailored by varying the crosslinking agent. The most common crosslinking agents are N-vinylpyrrolidone, which has the disadvantage that the unreacted monomer is toxic^8^, and methyl methacrylate^17^. He *et al*. used a poly(ethylene glycol) dimethacrylate (PEGDMA) as nontoxic crosslinking reagent in order to develop PPF hydrogels reinforced with β-tricalcium phosphate^18^.

Current efforts in the development of high performance biopolymer composites aim at increasing the dispersibility of the nanofillers and the polymer/nanofiller adhesion through covalent or non-covalent functionalization of the reinforcing agents. However, only very few studies reported the covalent functionalization of graphene oxide (GO) in order to increase its compatibility with biopolymer matrices^19^.

This study deals, for the first time, with the functionalization of GO with 2-hydroxyethyl methacrylate (HEMA). The vinyl monomer was covalently grafted through esterification onto the surface of carboxylated GO, as we anticipated that this surface modification approach facilitates the dispersion and improves the compatibility of the nanofiller with the PPF/PEGDMA matrix. Moreover, the vinyl double bond of HEMA can take part in the crosslinking process, thus improving the network properties of the hybrid materials.

Further, we examined systematically the effects of nanofiller incorporation on the properties of the crosslinked PPF/PEGDMA hybrid materials. Accordingly, the compressive strength and compressive modulus, morphology, equilibrium water content, degradation and mineralization behavior, as well as cytotoxicity were investigated. Additionally, the crosslinking characteristics of the hybrid materials were assessed by measuring the degree of swelling and sol fraction.

## Materials and Methods

Carboxylated graphene oxide (GO-COOH) with 0.7 mmol -COOH equivalents/g was purchased from NanoInnova. Diethyl fumarate, propylene glycol, hydroquinone, anhydrous ZnCl_2_, dichloromethane (DCM), acetone, tetrahydrofuran (THF), diethyl ether, anhydrous N,N-dimethylformamide (DMF), anhydrous Na_2_SO_4_, 1,1′-carbonyldiimidazole (CDI), 2-hydroxyethyl methacrylate (HEMA), 1,8-diazabicyclo[5.4.0]undec-7-ene (DBU), poly(ethylene glycol) dimethacrylate (PEGDMA) with the number average molecular weight (M_n_) of 750 g/mol, benzoyl peroxide (BP), N,N-dimethyl-*p*-toluidine (DMT), trifluoroethanol, N,N′-diisopropylcarbodiimide (DIC), pyridine (Py), bromine, were purchased from Sigma-Aldrichor Acros and used without further purification. Tris(hydroxymethyl)amino methane (Tris), NaCl, KCl, MgCl_2_, NaHCO_3_, CaCl_2_, KH_2_PO_4_, Na_2_HPO_4_, 37% HCl were purchased from Sigma-Aldrich or Merck and used as received.

### Synthesis of PPF

Poly(propylene fumarate) was synthesized following a protocol described in the literature^20^ and purified by precipitation in diethyl ether. The synthesized polymer was characterized by ^1^H-NMR, IR and GPC.

^1^H-NMR (300 MHz, CDCl_3_, δ, ppm): 1.10–1.18 (m, terminal -O-CH_2_-CH(C*H*_3_)-O-); 1.23 (d, backbone -O-CH_2_-CH(C*H*_3_)-O-); 3.54–3.58 (m, terminal -O-C*H*_2_-CH(CH_3_)-O-); 3.96–4.00 (m, terminal -O-CH_2_-C*H*(CH_3_)-O-); 4.10–4.27 (m, backbone -O-C*H*_2_-CH(CH_3_)-O-); 5.15–5.21 (m, backbone -O-CH_2_-C*H*(CH_3_)-O-); 6.72 (br. s, backbone *trans* -C*H*=C*H*-).

FT-IR (ATR, cm^−1^): 2986 (ν_C-H_), 1720 (ν_C=O_), 1645 (ν_C=C_), 1257, 1154 (ν_C-O_), 1154, 980 (δ_=C-H_).

GPC: M_n_ = 1400 g/mol; PDI = 1.85.

### Synthesis of GO@HEMA

In a 500 mL two-neck round-bottom flask, 250 mg of GO-COOH were added to 250 mL of anhydrous DMF. The flask was flushed with argon and sonicated in an ultrasonic bath for 4 hours at 20–25 °C. Then 1 g of CDI was added and the resulting mixture was sonicated again for 4 hours at 20–25 °C, transferred under argon to a stirred pressure filtration cell (Millipore XFUF04701) and filtered through a 0.2 μm Teflon membrane. The precipitate was washed with anhydrous DMF (3 × 25 mL), resuspended in 250 mL of anhydrous DMF and transferred under argon back into the round-bottom flask. The suspension was sonicated at 20–25 °C for 4 hours, a magnetic follower was placed in the flask followed by the addition of 1.62 g of HEMA and 1.9 mL of DBU catalyst and the suspension was stirred at room temperature for 24 h under argon. Finally, the mixture was transferred into the pressure filtration cell and GO@HEMA was filtered through a 0.2 μm Teflon membrane. The precipitate was washed with 2 × 25 mL DMF followed by2 × 25 mL acetone and dried at room temperature under vacuum (5 mmHg, 24 h).

### Synthesis of the hybrid materials

1.25 g of PPF and 1.25 g of PEGDMA were stirred until a homogenous mixture was obtained, then appropriate amounts of GO@HEMA were added in order to obtain a certain nanofiller concentration (0.5, 1 or 2 wt.%) and the resulting suspensions were sonicated using a probe for 30 min. at 5–10 °C. Then a solution of 7.5 mg BP in 0.1 ml DCM was added under stirring for 30 s followed by 3.75 µL DMT under rapid stirring for 10 s. Finally, the resulting mixtures were transferred to polypropylene vials and placed in a vacuum oven. Samples were kept under vacuum (10 mmHg) at room temperature for 2 h for degassing and removal of residual DCM, then the temperature was raised to 80 °C and the samples were allowed to cure for 24 h.

### Gas-phase chemical derivatization for XPS

- A sample of GO@HEMA placed in a small glass vial was inserted in a larger glass tube fitted with a Teflon stopper. Trifluoroethanol (0.45 mL) was carefully added in the outer tube, followed by 0.2 mL pyridine and 0.15 mL DIC. After 24 h at room temperature, the inner vial was taken out and transferred to the vacuum oven where it was kept at 5 mmHg for 24 h^21^.
- A sample of GO@HEMA placed in a small glass vial was inserted in a larger glass tube fitted with a Teflon stopper. Bromine (0.1 mL) was carefully added in the outer tube. After 4 h at room temperature, the inner vial was taken out and transferred to the vacuum oven where it was kept at 5 mmHg for 24 h^22^.

### Characterization

ATR FT-IR spectra were recorded on a Vertex 70 spectrometer (Bruker) in the 4000–600 cm^−1^ range, at a resolution of 4 cm^−1^. Raman spectra were acquired using an in Via confocal Raman microscope (Renishaw) equipped with a 473 nm excitation laser, at 0.4 mW incident power and a resolution of 2 cm^−1^. NMR spectra were recorded on a Fourier 300 spectrometer (Bruker). Gel permeation chromatography was performed using a PL-GPC 50 GPC/SEC System (Agilent) equipped with a Styragel column. THF (1 mL min^−1^) was employed as mobile phase and the calibration curve was generated with polystyrene standards.

TGA curves were registered on a Q500 thermobalance (TA Instruments), from room temperature to 900 °C, at a heating rate of 10 °C/min, under a dynamic nitrogen atmosphere.

The morphology of PPF/PEGDMA/GO@HEMA hybrid materials was studied using a Quanta Inspect F field emission scanning electron microscope (FEI) fitted with a detector for energy dispersive X-ray spectrometry (EDX). The diffraction patterns of the mineralized scaffolds were collected with a X’Pert Pro diffractometer (Panalytical) using CuKα radiation (λ = 1.5418 Å), over a range of 1°– 40° (2 θ), at a scan rate of 1°/min.

X-ray photoelectron spectroscopy (XPS) was performed on a K-Alpha spectrometer (Thermo Scientific), with monochromatic Al Kα X-rays (1486.6 eV). Sample charging was compensated by a flood gun and binding energies were calibrated by placing the C 1 s peak at 284.6 eV as internal standard. Survey and high-resolution spectra were recorded at resolutions of 200 eV and 20 eV, respectively. The deconvolution of core-level spectra with a mixed Gaussian-Lorentzian function was performed after a Shirley background subtraction.

Compression mechanical tests were performed according to the EN ISO 604:2002 standard, using a Model 3382 universal testing machine (Instron), at an ambient relative humidity of 45–50%. Dry cylindrical specimens having a diameter of 6 mm and a length of 12 mm were compressed along their long axis until failure, at a crosshead speed of 1 mm/min. The compressive modulus was determined as the slope of the linear portion of the stress-strain curve and the compressive strength was calculated by dividing the maximum compressive load carried by the specimen during the test by the initial cross-sectional area of the specimen. A minimum of five specimens were tested for each material composition and results are reported as average values ± standard deviation.

The *water uptake capacity* (*W*_*up*_) was determined gravimetrically. Weighed dry specimens were immersed in phosphate buffer saline (PBS) at 37 °C for 48 h, when the equilibrium water content was achieved. The samples were then removed from the solution, blotted with filter paper and weighed again. The water uptake capacity was calculated with the following formula:

1 $${W}_{up}( \% )=\frac{w-{w}_{0}}{{w}_{0}}\times 100$$

where *w* is the weight of the wet sample after immersion in PBS and *w*_0_ is the initial weight of the sample. All data are reported as average value ± standard deviation of three different measurements.

The *degree of swelling* (*DS*) and *sol fraction* (*SF*) were also determined following the above procedure except that toluene was used instead of PBS. This particular solvent was chosen due to the ability of PPF to swell moderately, so the samples maintain their 3D structure throughout the experiment^5^. In a typical experiment, dry specimens were weighed, placed into vials containing 20 ml of toluene and then stirred (100 rpm) at room temperature. After 24 h, the samples were taken out, blotted with filter paper and weighted. For the sol fraction determination, all samples were subsequently dried in the vacuum oven (37 °C, 10 mmHg) for 48 hours and weighed again. The sol fraction (Eq. 2) and degree of swelling (Eq. 3) were calculated using the formulas:

2 $$SF( \% )=\frac{{w}_{0}-{w}_{f}}{{w}_{0}}\times 100$$

3 $$DS( \% )=\frac{w-{w}_{0}}{{w}_{0}}\times 100$$

where *w*_*f*_ is the final weight of the dried sample after immersion in toluene, *w*_0_ is the initial weight of the sample before immersion and *w* is the weight of the wet sample, after immersion in toluene.

The non-enzymatic degradation of the hybrid materials was conducted by immersing the samples in PBS solution (pH 7.4) at 37 °C for 9 weeks. The solution was changed daily during the first week and then once a week. Samples were dried at 37 °C for 48 hours and weighed just before immersion. After immersion, samples were dried in the vacuum oven (37 °C, 10 mmHg) for 72 hours and weighed again. The *degradation weight loss percentage* (*WL*) was calculated using Eq. 4.

4 $$WL( \% )=\frac{{w}_{0}-{w}_{f}}{{w}_{0}}\times 100$$

where *w*_*f*_ is the final weight of the dried sample after immersion in PBS, and *w*_0_ is the initial weight of the sample.

#### Mineralization assay

The alternate soaking protocol developed by Taguchi *et al*. was employed in the mineralization study^23^. Briefly, samples cut into pieces of about 0.5 g each were soaked alternately for 24 h at 37 °C in two solutions: the first solution consisted in 200 mM CaCl_2_/Tris (pH adjusted to 7.4 with 0.5 N HCl) and the second solution consisted in 120 mM Na_2_HPO_4_. The alternate soaking cycle was repeated twice and then the samples were gently washed with distilled water to remove soluble salts and dried in the vacuum oven (37 °C, 10 mmHg) for 48 h.

#### Cell culture model

All tested composites were sterilized in advance by exposure to UV light. Then, murine pre-osteoblasts from MC 3T3-E1 (ATCC, CRL-2593) cell line were seeded at a density of 2 × 10^4^ cells/cm^2^ on the surface of the samples, resulting in a cell-scaffold system. This system was maintained for 6 days in Dulbecco’s modified Eagle medium (DMEM, Sigma-Aldrich) supplemented with 10% fetal bovine serum (FBS, Life Technologies) and 1% antibiotic (Sigma-Aldrich) under standard culture conditions (5% CO_2_, 37 °C and 95% relative humidity).

#### *In vitro* biocompatibility assessment

MTT assay: Cell viability and proliferation was assessed using methylthiazolyldiphenyl tetrazolium bromide (MTT, Sigma-Aldrich). The solution was prepared at the concentration of 1 mg/mL in culture media lacking FBS. After removal of complete media, the cell-scaffold systems were incubated for 4 h with MTT solution in the dark and standard culture conditions. The resulting blue-purple formazan crystals were dissolved in isopropanol and the absorbance of the resulting solution was measured at 550 nm using a FlexStation3 microplate reader (Molecular Devices).

LDH assay: For evaluating the materials’ cytotoxicity, a commercial “Lactate dehydrogenase based *in vitro* toxicology assay kit” (TOX7 kit, Sigma-Aldrich) was used. Solutions were prepared following the manufacturer’s instructions and the absorbance of the final assay solution was measured at 490 nm using a Flex Station 3 microplate reader (Molecular Devices).

All biocompatibility experiments were performed in triplicate (n = 3) and results were assessed using one-way ANOVA method and Bonferroni post-test. Results were expressed as mean value ± standard deviation (SD) using GraphPad Prism Software 3.0 (GraphPad Software Inc.).

## Results and Discussions

### Synthesis and characterization of GO@HEMA

**Figure 1:**
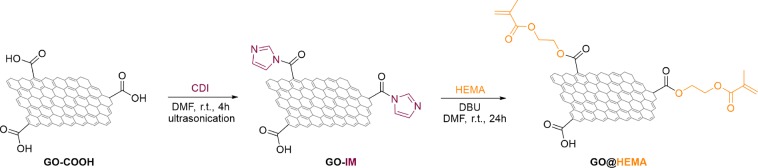
Synthesis of GO@HEMA. The structures of carboxylated GO flakes are simplified, as the scheme is meant to illustrate only the esterification reaction with HEMA.

A two step protocol was developed for the functionalization of commercially available carboxylated graphene oxide (GO-COOH). In the first step, surface carboxymethyl groups were activated with N,N-carbonyldiimidazole (CDI)^24,25^ to form an intermediate imidazolide-functionalized GO (GO-IM) (Fig. 1). In the second step, excess CDI was removed by filtration and washing, the intermediate GO-IM was resuspended in anhydrous DMF and further reacted with 2-hydroxyethylmethacrylate (HEMA). In the second step, DBU was employed as catalyst for the esterification reaction because the reactivity of acyl imidazole groups is lower than the corresponding acid chlorides, which are the common intermediates for esterification of surface carboxylate groups^26,27^.

**Figure 2:**
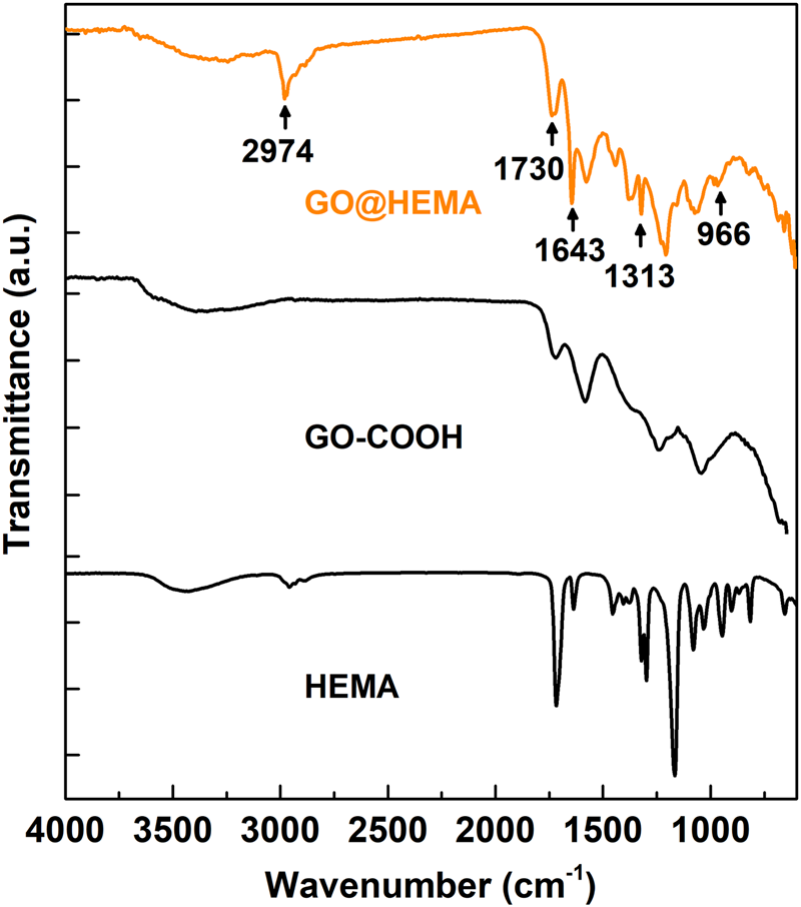
FT-IR spectra of HEMA, GO-COOH and GO@HEMA. In the spectrum corresponding to GO@HEMA, the bands at 966 cm^−1^, 1313 cm^−1^, 1643 cm^−1^ and 2974 cm^−1^ suggest the presence of surface-grafted HEMA molecules.

The functionalization of GO-COOH with HEMA was first monitored using FT-IR spectroscopy, and Fig. 2 exhibits the spectra of GO-COOH,GO@HEMA and HEMA. GO-COOH shows four bands at 1718 cm^−1^, 1583 cm^−1^, 1240 cm^−1^ and 1041 cm^−1^, the characteristic band at 1718 cm^−1^being unambiguously attributed to the C=O stretching vibration of carboxylic groups^28^. Compared with the GO-COOH spectrum, the spectrum of GO@HEMA exhibits several new bands, at 966 cm^−1^, 1313 cm^−1^, 1643 cm^−1^, 2974 cm^−1^. These bands suggest the presence of surface-grafted HEMA molecules and were attributed to the alkene = C-H bending vibration, ester C-O stretch, alkene C=C stretch and aliphatic C-H stretching vibration, respectively^29^. Moreover, the C=O stretching band at 1718 cm^−1^ is shifted to 1730 cm^−1^, indicating the presence of α,β-unsaturated ester groups.

**Figure 3:**
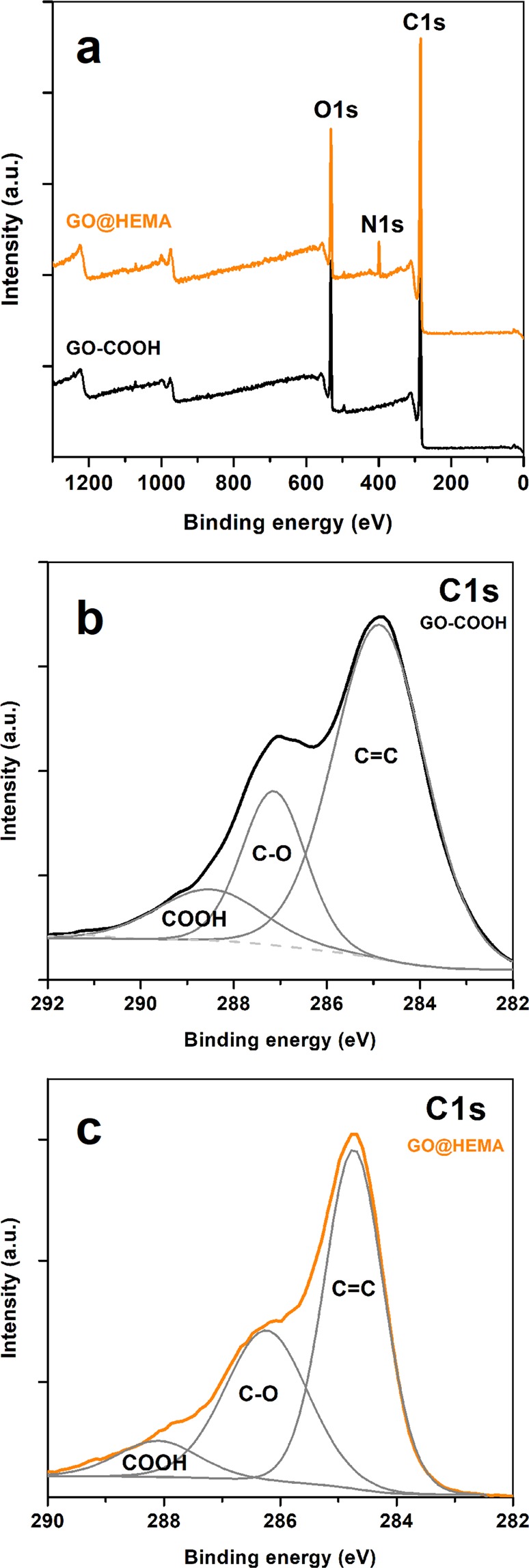
XPS survey spectra of GO-COOH before and after functionalization with HEMA (**a**), and C1s core-level spectra of carboxylated GO (**b**) and GO@HEMA. **(c**) In case of GO@HEMA, the C=C/C-O ratio decreases from 3.1 to 1.1 and the C=C/COOH ratio decreases from 5.7 to 4.0, indicating that a fraction of the carboxyl groups have reacted with HEMA.

A more in-depth analysis of the surface chemistry of GO@HEMA was performed using XPS. The survey spectra of GO-COOH and GO@HEMA presented in Fig. 3a reveal the presence of C1s and O1s peaks; additionally, the N1s peak appearing in the spectrum of GO@HEMA can be attributed to unreacted acyl imidazole groups. Further, the C1s core-level spectra (Fig. 3b,c) registered for GO-COOH and GO@HEMA were deconvoluted into three components positioned at 284.8 eV, 287 eV, 288 eV, which correspond to carbon atoms from C=C double bonds, C-O single bonds (alcohol, phenol or epoxide groups), and carboxyl groups, respectively^30^. In case of GO@HEMA, the C=C/C-O ratio decreases from 3.1 to 1.1 and the C=C/COOH ratio decreases from 5.7 to 4.0, indicating that a fraction of the carboxyl groups have reacted with HEMA.

**Figure 4:**
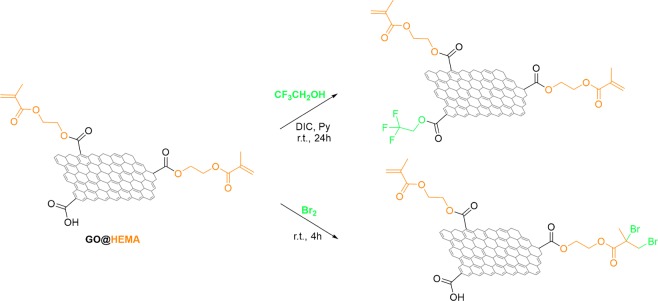
Gas-phase chemical derivatization of GO@HEMA for XPS analysis. The structures of GO@HEMA flakes are simplified, as the scheme is meant to illustrate only the most plausible reactions with the derivatization reagents.

The use of chemical derivatization allows for a better understanding of the changes in surface chemistry of graphene materials than direct XPS analysis and for this purpose, two gas-phase derivatization reactions were chosen in this study (Fig. 4). For the analysis of surface carboxyl functionalities, trifluoroethanol (TFE) was employed as derivatization reagent in conjuction with a carbodiimide reagent^21,31^.

**Figure 5:**
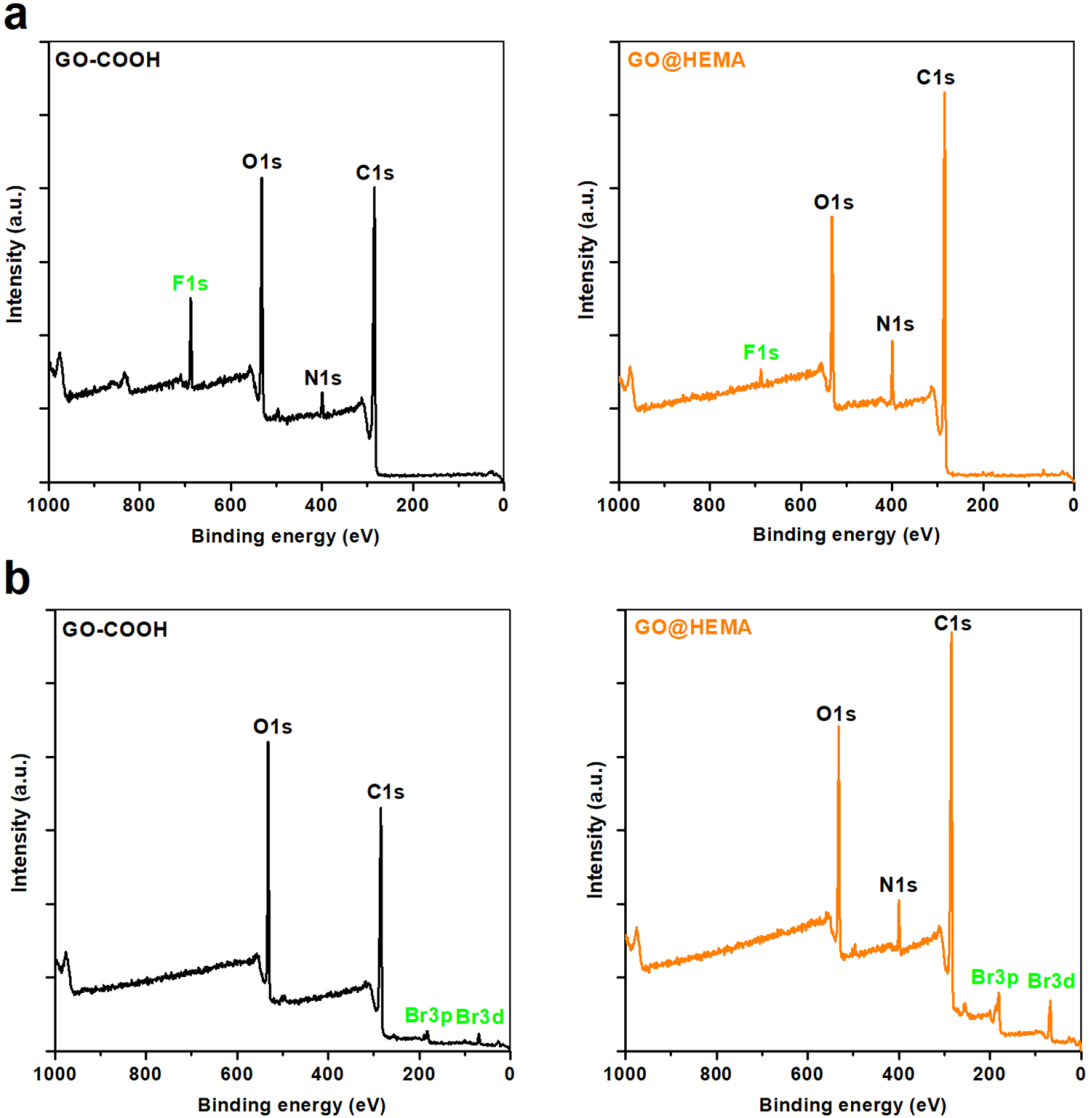
XPS survey spectra of GO-COOH and GO@HEMA after chemical derivatization with trifluoroethanol (**a**) and bromine. (**b**). The C/F ratio for GO@HEMA is almost 6 times larger as compared to GO-COOH, a strong indication that most of the carboxyl groups are esterified with HEMA. The C/Br ratio for GO@HEMA is almost 3 times lower as compared to GO-COOH, suggesting a significantly larger amount of bromine added to the C=C bond in HEMA and thus providing further evidence for the surface grafting of the monomer molecule.

The appearance of F1s peaks in the survey scans of TFE-derivatized GO-COOH and GO@HEMA indicates the presence of carboxylic acid functionalities (Fig. 5). Compared with GO-COOH, the C/F ratio for GO@HEMA increases almost 6 times, a strong indication that most of the carboxyl groups are esterified with HEMA and therefore unreactive towards TFE (transesterification reactions are unlikely to occur here). For the analysis of alkenyl surface groups, a gas-phase bromination reaction was used, as it is known that under these conditions bromine adds to double bonds in unsaturated carboxylic acid derivatives^22^. The appearance of bromine peaks in the survey scans of Br_2_-derivatized GO-COOH and GO@HEMA can be explained by intercalation of molecular Br_2_ between the graphene sheets, presumably forming C_n_-Br_2_ charge-transfer complexes^32^ or by a reaction with surface carboxyl groups to form carboxyl bromides^33^. However, in case of GO@HEMA, the C/Br ratio is almost 3 times lower, suggesting a significantly larger amount of bromine added to the C=C bond in HEMA and thus providing further evidence for the surface grafting of the monomer molecule.

**Figure 6:**
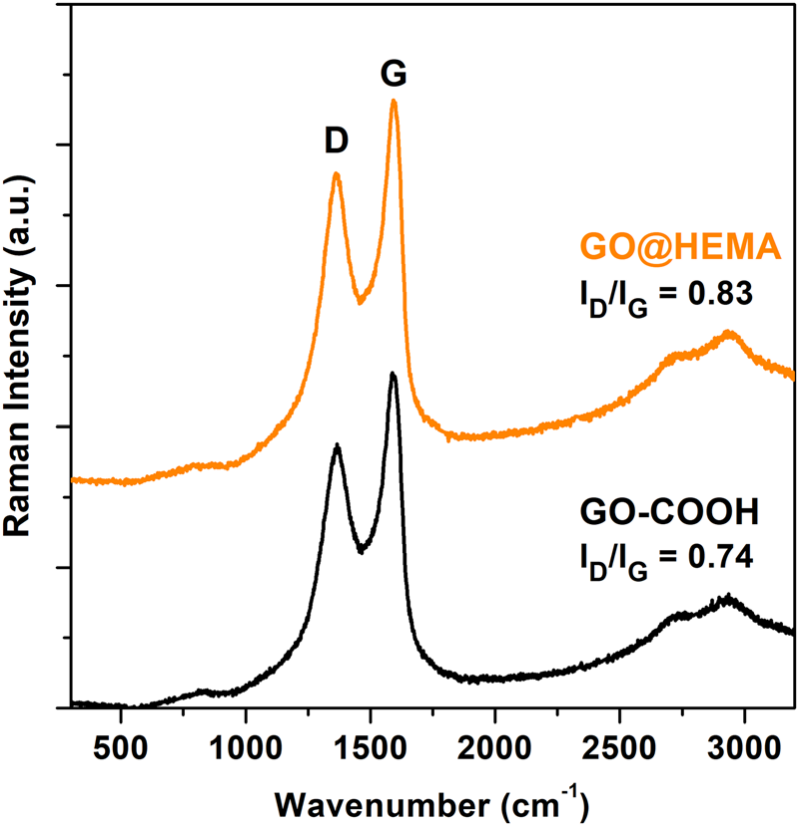
Raman spectra of GO-COOH and GO@HEMA. The similar I_D_/I_G_ ratios indicate that functionalization with HEMA doesn’t induce more defects in the graphene lattice.

The Raman spectra recorded for GO-COOH and GO@HEMA (Fig. 6) exhibit the two characteristic peaks of graphitic carbon materials, the G-band at 1580 cm^−1^ and the D-band 1350 cm^−1^, assigned to the sp^2^-hybridised carbon system and defects in the graphene layer, respectively^28^. In order to obtain quantitative information about the amount of defects induced by the chemical modification process, the I_D_/I_G_ ratio was calculated. As expected, the values reported in Fig. 6 are close, indicating that our modification protocol did not induce more defects in the graphene lattice, since the monomer is linked by ester groups to carboxylic functionalities already present in the starting material.

**Figure 7:**
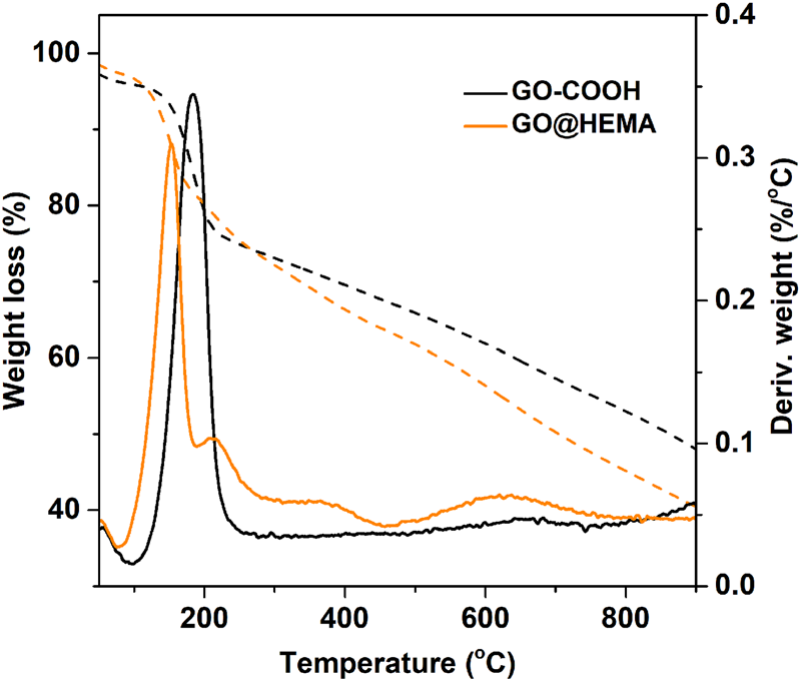
TGA (solid line) and DTG (dashed line) curves of GO-COOH and GO@HEMA illustrating a decrease of the residual weight in the case of GO@HEMA as compared with GO-COOH.

Additional information regarding the functionalization reaction, although indirect, was revealed through thermogravimetric analysis. The TGA curves of GO-COOH and GO@HEMA (Fig. 7) display two degradation steps: one below 100 °C, which is attributed to water evaporation, and a second one at 660 °C, assigned to the pyrolysis of surface functional groups and degradation of the carbon skeleton. Compared to pristine GO-COOH, GO@HEMA shows additional degradation processes at 210 and 360 °C accompanied by a significant decrease in residual weight, denoting the presence of grafted HEMA^34^.

### Synthesis and characterization of PPF/PEGDMA/GO@HEMA hybrid materials

Although poly(propylene fumarate) is a valuable polymer and its use in tissue engineering applications has been well documented, it is not available commercially. Accordingly, PPF was synthesized following a method described in the literature^20^. In the first step, the intermediate bis(2-hydroxypropyl fumarate) was obtained from diethyl fumarate and propylene glycol by ZnCl_2_-catalyzed transesterification (140 °C, 8 h). The polymer was synthesized in the second step, through polycondensation of the intermediate at high temperature and under vacuum (130 °C, 10 mmHg, 8 h). Poly(ethylene glycol) dimethacrylate was used as crosslinking agent for the hybrid materials containing GO@HEMA. The double-bond ratio between PPF and PEGDMA chosen for this study was 1:1^18^.

**Figure 8:**
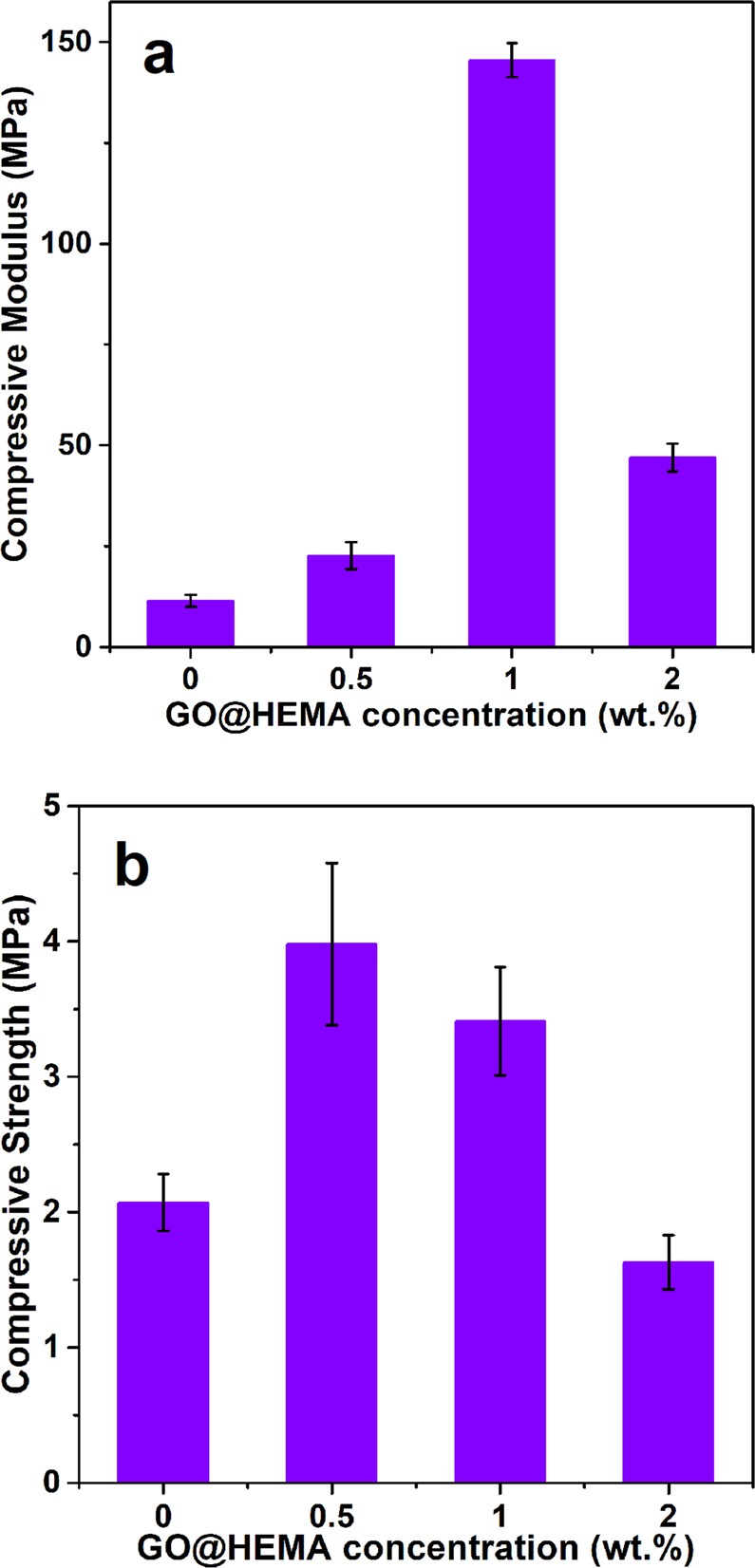
The compressive modulus (**a**) and compressive strength (**b**) of neat PPF/PEGDMAand hybrid materials containing various GO@HEMA loadings. The hybrid materials show a 14-fold improvement of the compressive modulus at 1 wt.% and 2-fold improvement of the compressive strength at 0.5 wt.% GO@HEMA loading, respectively.

Considering that mechanical properties are essential for medical applications such as bone repair, the compressive strength and compressive modulus of neat PPF/PEGDMA and PPF/PEGDMA/GO@HEMA hybrid materials were measured and the corresponding results are reported in Fig. 8. Crosslinked PPF/PEGDMA exhibits a compressive modulus of about 11 MPa and the introduction of GO@HEMA leads to an outstanding enhancement of the compressive modulus, which shows a 14-fold improvement at 1 wt.% GO@HEMA loading. This significant modulus increase can have several explanations, the most straightforward being the good reinforcement effect of GO@HEMA due to its good compatibility and strong adhesion with the polymer matrix, combined with a high intrinsic modulus of GO^35^. Another reason for this improvement might be the presence of vinyl double bonds on the surface of GO@HEMA, which can take part in the crosslinking process resulting in a higher crosslinking density^15^. However, for the composite containing the highest amount of GO@HEMA the compressive modulus decreases, and this was attributed to the agglomeration of nanofiller.

Regarding the compressive strength, a 2-fold enhancement can be observed at 0.5 wt.% GO@HEMA and then the compressive strength decreases with increasing nanofiller amount, reaching at 2 wt.% GO@HEMA even a smaller value than neat PPF/PEGDMA. The enhancement in compressive strength for the hybrid material containing a small amount of GO@HEMA suggests that a homogenous dispersion of highly exfoliated nanofiller was obtained at this concentration. On the contrary, GO@HEMA has a tendency to agglomerate at higher concentration, as evidenced by the SEM results reported below. The negative outcome obtained for the composite with the highest amount of GO@HEMA can therefore be explained by the fact that agglomerated GO@HEMA flakes act as defects in the material, accelerating the propagation of cracks^13^. This tendency of nanofillers to agglomerate at higher concentration leading to a decrease in mechanical properties has also been described in several other studies^36,37^.

Considering the reported values, we can conclude that the mechanical properties of the synthesized hybrid materials, i.e. a compressive modulus of 145.5 ± 4.1 MPa and compressive strength of 3.4 ± 0.4 MPa, meet the design goals for bone substitutes, being close to those of human trabecular bone^38^.

The high mechanical reinforcement of the hybrid materials is further supported by an investigation of their network properties. Both the degree of swelling (DS) and sol fraction (SF) were determined in order to study the interaction of GO@HEMA with PPF/PEGDMA during the crosslinking process, as a decrease in DS and SF indicates that more double bonds are involved in crosslinking, leading to denser networks with improved mechanical properties^16^. According to Table 1, DS decreases from 38.0 ± 0.1% to 19.0 ± 0.5% with increasing GO@HEMA content. The trend is similar for SF, except for the material containing the highest amount of GO@HEMA, presumably due to agglomeration of the nanofiller. These results support our initial assumption that vinyl double bonds on the surface of GO@HEMA are involved in the crosslinking process, leading to an increased crosslinking density in the hybrid materials.

**Table 1:** The degree of swelling in toluene, sol fraction, water uptake capacity and degradation weight loss for crosslinked PPF/PEGDMA and hybrid materials with various GO@HEMA loadings.

Sample	Degree of swelling in toluene (%)	Sol fraction in toluene (%)	Water uptake capacity in PBS (%)	Degradation weight loss in PBS (%)
PPF/PEGDMA	38.0 ± 0.1	35.7 ± 0.2	28.7 ± 0.8	3.5 ± 0.2
PPF/PEGDMA/GO@HEMA 0.5%	29.8 ± 0.6	28.3 ± 0.5	28.6 ± 0.6	6.6 ± 0.7
PPF/PEGDMA/GO@HEMA 1%	26.3 ± 0.2	27.9 ± 0.4	31.2 ± 0.1	7.9 ± 0.6
PPF/PEGDMA/GO@HEMA 2%	19.0 ± 0.5	30.8 ± 0.2	30.1 ± 0.6	17.0 ± 0.3

The water uptake capacity of neat PPF/PEGDMA is 28.7 ± 0.8%, similar with values reported in the literature for PPF crosslinked with PEGDMA^18^. The incorporation of GO@HEMA within the polymer matrix seems to have no significant influence on the equilibrium water content of the PPF/PEGDMA/GO@HEMA networks.

**Figure 9:**
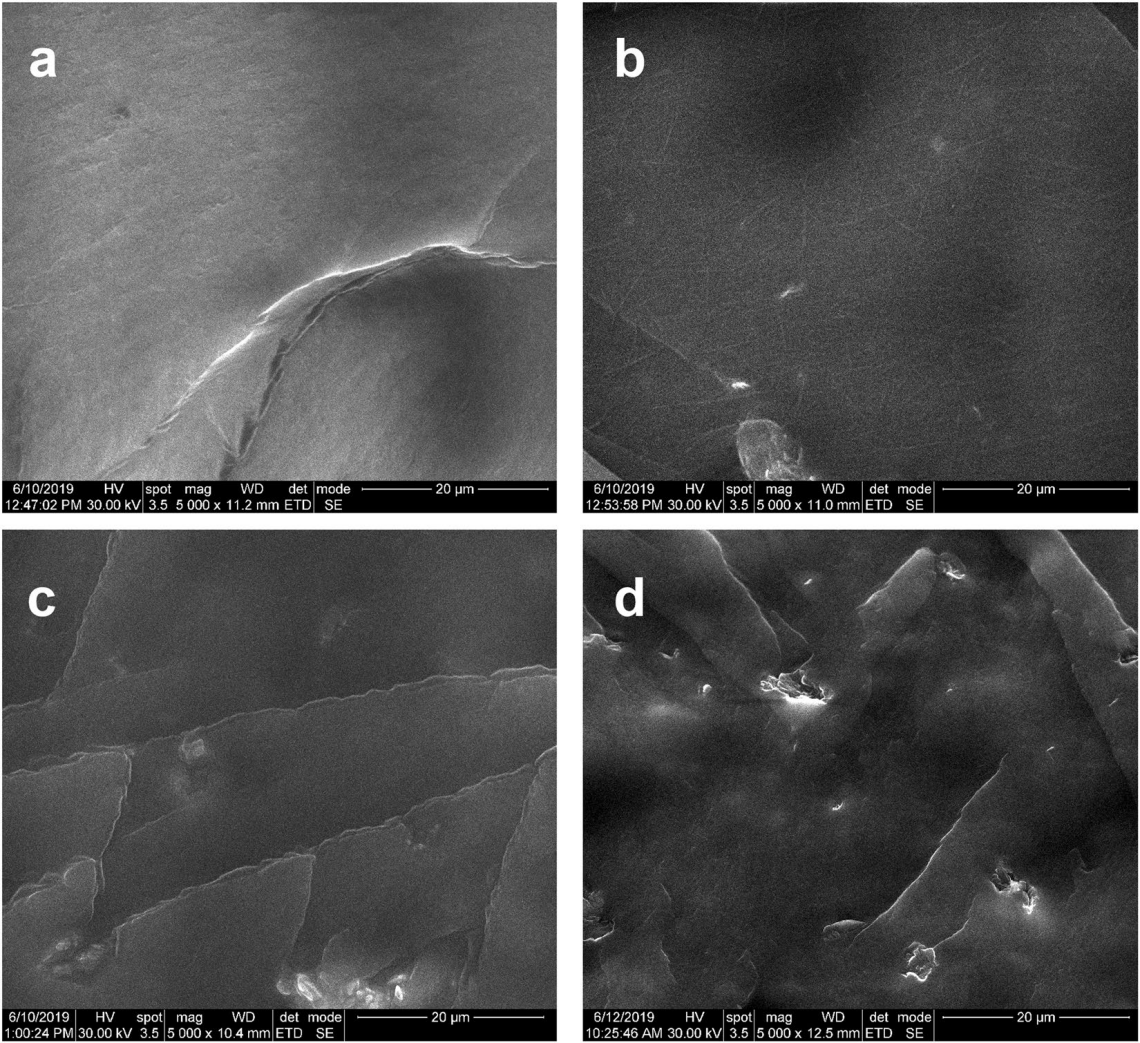
SEM micrographs of PPF/PEGDMA **(a**) and PPF/PEGDMA/GO@HEMA containing 0.5 wt.% (**b**), 1 wt.% (**c**) and 2 wt.% (**d**) GO@HEMA loadings. While the image corresponding to PPF/PEGDMA/GO@HEMA with 0.5 wt.% GO@HEMA exhibits a random and homogenous dispersion of nanofiller flakes (**a**), a tendency to form aggregates is observed at 1 and 2 wt.% (**c**,**d**).

A SEM investigation was performed in order to better understand the dispersion state of the GO@HEMA nanofiller inside the PPF/PEGDMA matrix. According to Fig. 9a, neat PPF/PEGDMA displays a smooth cross-section morphology. At 0.5 wt.% concentration, GO@HEMA flakes exhibit a random and homogeneous dispersion in the polymer matrix (Fig. 9b). By further increasing the GO@HEMA loading to 1 and 2 wt. %, the micrographs (Fig. 9c,d) reveal a rough cross-section with some agglomeration of nanofiller. These observations appear to support our results regarding the mechanical properties, because the nanofiller dispersion inside the polymer matrix plays an important role in the storage and loss of applied mechanical energy^39^. Therefore, GO@HEMA concentrations lower than 1% seem more appropriate for developing high performance hybrid materials.

Another important factor which should be considered when selecting materials for tissue engineering is their biodegradability, and ideally the degradation rate must closely match the rate of tissue regeneration^40^. Thus, the *in vitro* non-enzymatic degradation of the synthesized materials was assessed through immersion in a PBS solution at 37 °C for 9 weeks, and the obtained results are summarized in Table 1. Neat PPF/PEGDMA displays a degradation weight loss of 3.5 ± 0.2%. For the hybrid materials containing GO@HEMA the weight loss is increased, reaching a value of 17.0 ± 0.3% at 2% GO@HEMA content. This suggests that the high acidity of GO^41^ is responsible for the increased hydrolysis rate of ester bonds within the polymer chains^42^. Similar results were reported for other biodegradable polymer composites such as poly(L-lactide)/GO^43,44^.

**Figure 10:**
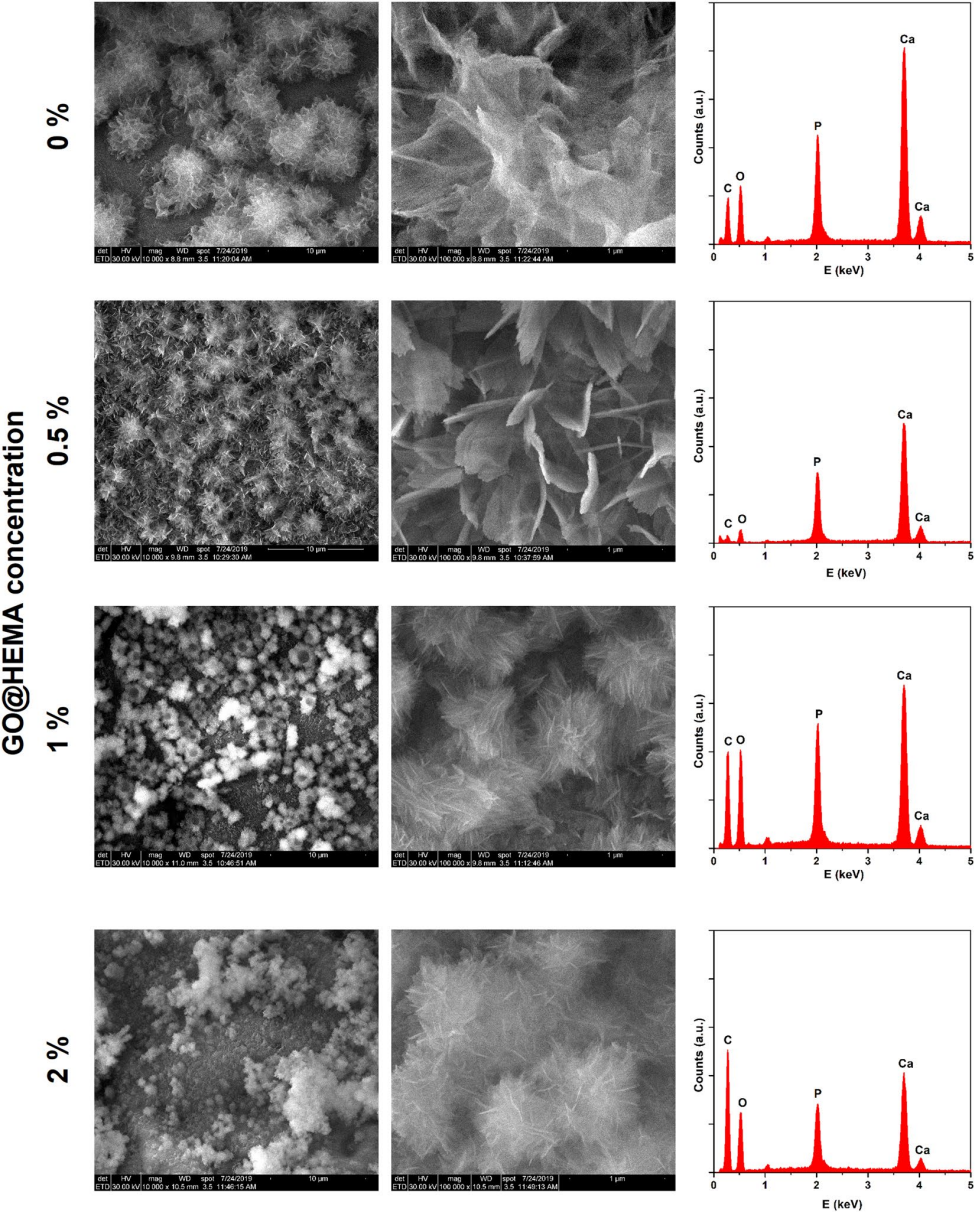
SEM micrographs at a magnification of 10 000 × (left), 100 000 × (middle) and typical EDX spectra of mineralized PPF/PEGDMA and PPF/PEGDMA/GO@HEMA hybrid materials (right). The micrographs show that all samples are covered with a continuous mineral phase displaying a spherulitic micro-morphology with agglomerations of plate-like crystals. Both the spherulite size and the crystal size decrease with increasing GO@HEMA content.

**Figure 11:**
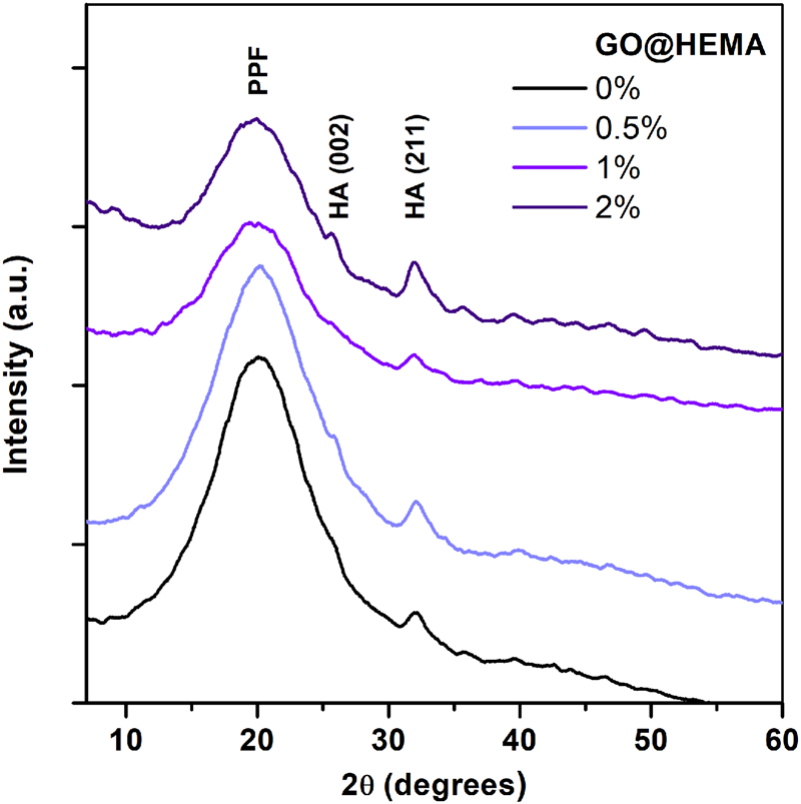
XRD patterns of PPF/PEGDMA and PPF/PEGDMA/GO@HEMA hybrid materials after mineralization. The diffraction peaks at 2θ ~ 26° and 32° are characteristic for the hexagonal hydroxyapatite phase.

Because the capacity of biomaterials to form bone-like hydroxyapatite (HA) surface layers is also essential for bone tissue engineering applications^45^, the *in vitro* bioactivity of the synthesized hybrid materials was evaluated through alternate soaking in solutions containing Ca^2+^ and HPO_4_^2−^. A SEM examination revealed that all mineralized samples are homogenously covered with a continuous mineral phase (Fig. 10). While neat PPF/PEGDMA shows a spherulitic micro-morphology with agglomerations of plate-like crystals, the addition of GO@HEMA within the polymer matrix clearly affects the morphology of the mineral phase, as the spherulites decrease in size and the crystal size also decreases. Apparently, at higher GO@HEMA content, the number of nucleation sites increases, leading in the same time to a decrease in nanocrystal size because the growth process is diffusion-controlled. This type of effect induced by graphene nanofillers was reported previously and it was alleged that the predominant sites for HA nucleation are the edges and the fractures of GO flakes^46,47^. Another plausible explanation is that HA nucleation is influenced by rugosity^48^, and in our case the SEM results previously discussed revealed that surface roughness of the hybrid materials increases with GO@HEMA loading. The chemical composition of the mineral phase was further evaluated by calculating the Ca/P ratio from EDX spectra. The Ca/P ratio is 1.70 for neat PPF/PEGDMA, 1.60 for 0.5 wt.% GO@HEMA, and decreases to 1.38 in case of the hybrid materials containing 1 and 2 wt.% GO@HEMA. These values are common for bone-like HA deposits obtained through *in vitro* mineralization^49^. The XRD patterns of mineralized samples (Fig. 11) display two peaks at 2θ~26° and 32°, characteristic for the hexagonal hydroxyapatite phase (JCPDS 00-09-0432), and a broad peak at about 20° assigned to the polymer. Based on these results it can be concluded that GO@HEMA influences the hybrid materials’ bioactivity, and its concentration can be fine-tuned in order to obtain bone-like HA coatings with appropriate morphology and Ca/P ratio.

**Figure 12:**
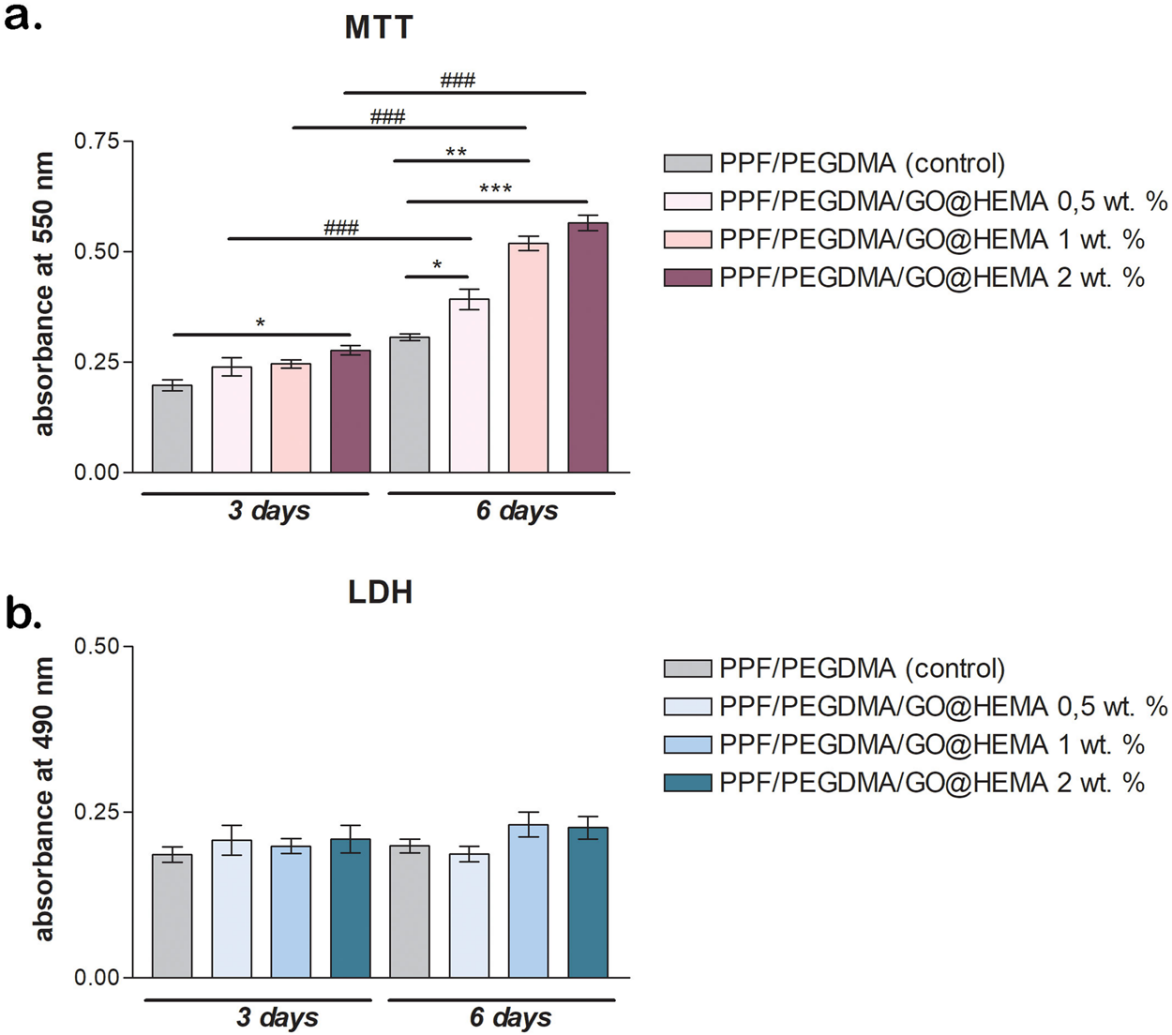
(**a**) Murine pre-osteoblasts viability and proliferation profiles resulting from the MTT assay after 3 and 6 days of *in vitro* cell culture (statistical significance: *p < 0.05; **p < 0.01; *** and ^###^p < 0.001) and (**b**) cytotoxicity evaluation by LDH assay during 6 days of *in vitro* cell culture. The MTT and LDH assays show that murine pre-osteoblasts maintained viability and started to proliferate from 3 to 6 days of culture and the incorporation of GO@HEMA within the polymer matrix did not induce any significant cytotoxic effects.

The cell viability and proliferation assessment using an MTT assay indicated that the majority of seeded cells displayed a good interaction with the hybrid materials, as the addition of GO@HEMA had a positive impact on cell viability and metabolic activity (Fig. 12). After 3 days of culture, no significant differences in viability were found in systems with 0.5% and 1% GO@HEMA as compared to the neat PPF/PEGDMA control. On the other hand, if the control was compared to the material enriched with 2% GO@HEMA, cell viability was significantly (p < 0.05) higher in the later case.

In contrast, at 6 days post-seeding, cell viability changed significantly (p < 0.05) in contact with scaffolds containing 0.5% GO@HEMA as compared to the control and also a significantly (p < 0.01) higher amount of viable cell was found on the hybrid material with 1% GO@HEMA. After 6 days of culture, the most significant (p < 0.001) viability level of murine pre-osteoblasts was found in contact with the material enriched with 2% GO@HEMA. The increased surface roughness of the hybrid material containing the largest amount of nanofiller might explain these observations, as cell adhesion and proliferation improve with increasing surface roughness^8^. Therefore, cell behavior changed due to the presence of GO@HEMA, indicating that the nanofiller had an important contribution in increasing and possibly supporting the metabolic activity of the cells. The MTT test also revealed that cells maintained viability and started to proliferate from 3 to 6 days of culture. Significant (p < 0.001) proliferation rate of murine pre-osteoblasts was found on all composites with GO@HEMA from 3 to 6 days, as compared to the control where no significant differences were observed.

Further, after performing an LDH assay it was concluded that none of the tested composites exhibited any cytotoxic effects on the murine cell line. Low levels of toxicity were found only at 3 days post-seeding, but they were not significant and, more important, the addition of GO@HEMA didn’t cause significant cell death. Interestingly, these levels remained constant throughout 6 days of culture, thus demonstrating that PPF/PEGDMA/GO@HEMA hybrid materials didn’t present toxic effects upon cells on a longer term.

## Conclusions

In this study, we report the synthesis and characterization of novel PPF/PEGDMA hybrid materials containing GO grafted with HEMA monomer moieties. The GO@HEMA nanofiller was designed in order to enhance the compatibility between GO and the PPF/PEGDMA polymer matrix and simultaneously augment the network properties of the resulting hybrid materials. The covalent modification of carboxylated GO with HEMA was successfully achieved through a 1,1′-carbonyldiimidazole-mediated esterification reaction. The incorporation of GO@HEMA into PPF/PEGDMA led to hybrid materials with considerably improved mechanical properties and this was partially attributed to an increased crosslinking density, evidenced by the degree of swelling and sol fraction values. We also demonstrated that GO@HEMA improves the biodegradability and influences the bioactivity of the hybrid materials, as the nanofiller concentration can be fine-tuned in order to obtain bone-like hydroxyapatite phases with appropriate morphology and Ca/P ratio. Finally, all synthesized hybrid materials were biocompatible with murine pre-osteoblasts and, more important, no cytotoxic effects were evidenced *in vitro*. All these results suggest that PPF-based hybrid materials containing GO@HEMA have a great potential for applications in the field of bone tissue engineering, and currently our efforts are focused on finding a suitable solution to increase the porosity of these materials, while preserving their excellent mechanical properties.
